# Capsular Ligament Function After Total Hip Arthroplasty

**DOI:** 10.2106/JBJS.17.00251

**Published:** 2018-07-18

**Authors:** Richard J. van Arkel, K.C. Geoffrey Ng, Sarah K. Muirhead-Allwood, Jonathan R.T. Jeffers

**Affiliations:** 1 Department of Mechanical Engineering, Imperial College London, London, United Kingdom; 2 The London Hip Unit, London, United Kingdom

## Abstract

**Background:**

The hip joint capsule passively restrains extreme range of motion, protecting the native hip against impingement, dislocation, and edge-loading. We hypothesized that following total hip arthroplasty (THA), the reduced femoral head size impairs this protective biomechanical function.

**Methods:**

In cadavers, THA was performed through the acetabular medial wall, preserving the entire capsule, and avoiding the targeting of a particular surgical approach. Eight hips were examined. Capsular function was measured by rotating the hip in 5 positions. Three head sizes (28, 32, and 36 mm) with 3 neck lengths (anatomical 0, +5, and +10 mm) were compared.

**Results:**

Internal and external rotation range of motion increased following THA, indicating late engagement of the capsule and reduced biomechanical function (p < 0.05). Internal rotation was affected more than external. Increasing neck length reduced this hypermobility, while too much lengthening caused nonphysiological restriction of external rotation. Larger head sizes only slightly reduced hypermobility.

**Conclusions:**

Following THA, the capsular ligaments were unable to wrap around the reduced-diameter femoral head to restrain extreme range of motion. The posterior capsule was the most affected, indicating that native posterior capsule preservation is not advantageous, at least in the short term. Insufficient neck length could cause capsular dysfunction even if native ligament anatomy is preserved, while increased neck length could overtighten the anterior capsule.

**Clinical Relevance:**

Increased understanding of soft-tissue balancing following THA could help to prevent instability and improve early function. This study illustrates how head size and neck length influence the biomechanical function of the hip capsule in the early postoperative period.

Many surgical approaches for total hip arthroplasty (THA) aim to maximize capsule preservation^1-10^ and/or repair capsule incisions^1-9^, while others excise the capsule to improve exposure, as it is considered of little consequence^11,12^. Capsule preservation and repair can help to lower dislocation rates^6,7,10,13-18^, and may also maintain the defenses of the native hip against hypermobility, impingement, subluxation, and edge-loading^19-22^.

Preserving a capsular ligament increases surgical complexity: it requires more precise incision, and it limits exposure. It is justified under the assumption that preserving native anatomy preserves biomechanical function. However, THA geometry is different from that of the native hip. Changes in femoral neck length and offset may affect ligament tensions and function^23^, and the smaller THA head size may inhibit ligament wrapping around the femoral head, a function that is vital to capsular biomechanics in the native hip (Fig. 1). However, to our knowledge these capsular function changes have never been measured after THA, so it is unknown how either THA per se, or operative variables of head size and neck length, affect capsule performance.

**Fig. 1:**
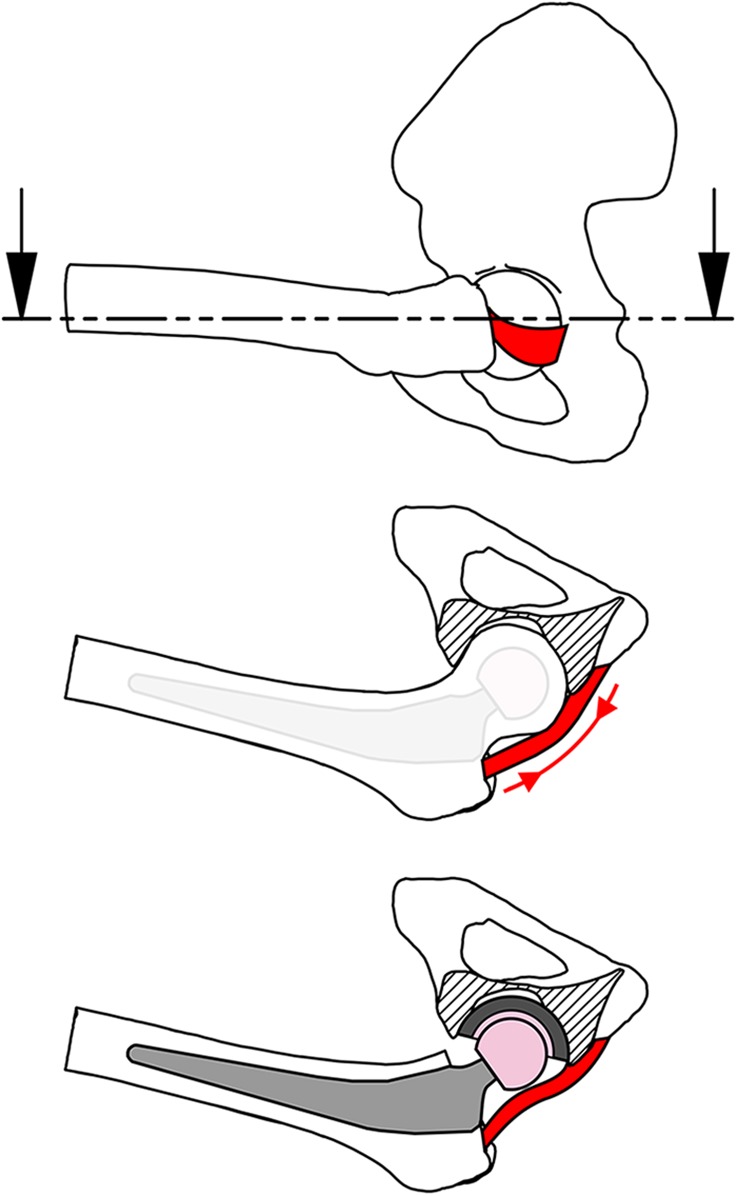
Drawings of a hip in deep flexion, with the ischiofemoral capsular ligament shown as the red band. Top: Sagittal view of the native hip, with the dashed line and arrows indicating a transverse section plane. Middle: Transverse section view showing the ligament taut, wrapped around the native femoral head, and supporting it in a sling. This tension (red arrows) provides restraint against excessive hip movement. A femoral component is shown faintly to indicate its reduced head size. Bottom: In the same hip position following arthroplasty, the ligament is slack as the reduced head size prevents the wrapping mechanism. There is no tension to resist extreme range of motion and no sling protecting against subluxation.

The present study used a specialty in vitro (nonclinical) THA preparation that preserved all of the capsular ligaments, in order to provide baseline data on how THA affects the early postoperative function of those ligaments. It was hypothesized that THA would adversely influence ligament biomechanics—i.e., manifest as hypermobility—and we investigated whether such influence could be moderated by pretensioning the ligaments via increasing the head size or neck length.

## Materials and Methods

### Testing Protocol

**Fig. 2:**
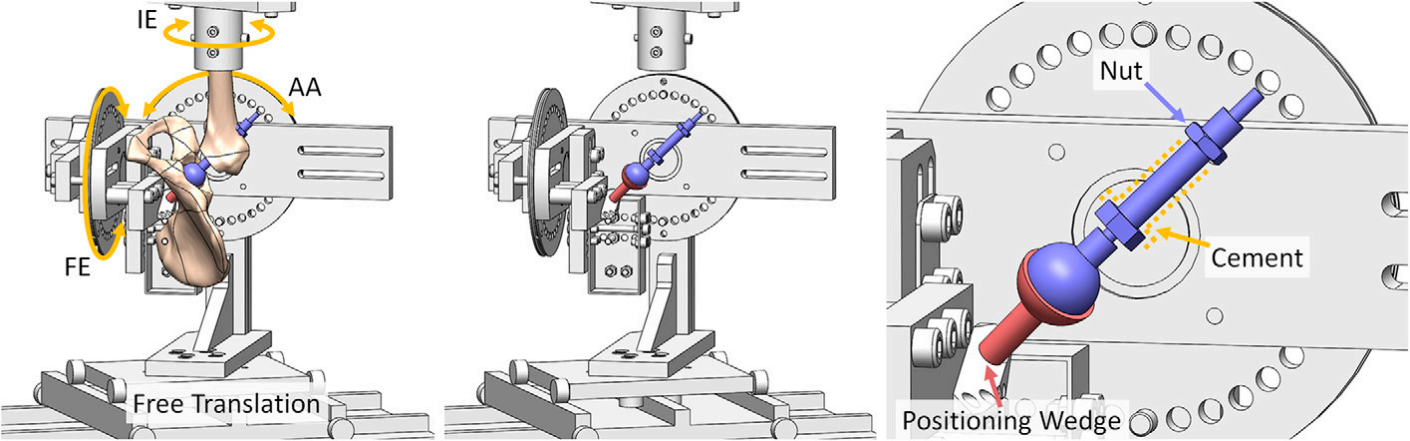
The testing rig setup following THA. Left: The full setup showing the flexion-extension (FE), abduction-adduction (AA), and internal-external rotation (IE) axes of movement. Middle: The same image but with the pelvis and femur hidden to enable direct visualization of the THA components. Right: An enlarged image of the THA components. The acetabular component is attached to the rig through the medial wall opening. Its orientation was set by the wedge component that rigidly attached it to the pelvis testing rig. The femoral component was positioned by the guidewire technique. It was fixed through a combination of bone cement and tightening the indicated nut.

After local research ethics committee approval was received, 8 fresh-frozen cadaveric hips (from 5 male and 3 female donors, with a mean age of 64 years [range, 48 to 82 years] at the time of death) were defrosted and skeletonized, carefully preserving the capsule. The hips were mounted into a 6-degrees-of-freedom testing rig (Fig. 2) using an established protocol^24^. This rig adopted the International Society of Biomechanics coordinate system^25^ and allowed either for flexion-extension or abduction-adduction torques to be applied through pulleys with hanging weight couples, or for these axes to be fixed at specific angular positions with screw clamps^19,22^. Internal-external rotations were applied using the rotating axes of a dual-axis servohydraulic materials testing machine (model 8874; Instron) equipped with a 2-degrees-of-freedom (tension-torque) load-cell, thus allowing the passive restraint to hip rotation from the capsular ligaments to be measured^19,22^. Translations were free to occur in response to applied load and/or movement^19,22^.

For each specimen, the hip joint center was registered to the rig by moving the hip throughout its range of motion and iteratively repositioning it until the error between the functional hip center and the rig center was <1 mm. The envelope of passive motion allowed by the capsular ligaments was then found using an established protocol, by sequentially applying 5-Nm torque about the desired movement axis while preventing rotation about the other axes^19,22^. First, a 5-Nm torque was applied to determine the limits of flexion-extension, and then of abduction-adduction, with the hip held in full flexion and in full extension. These limits of flexion-extension and abduction-adduction were used for all subsequent tests of the native hip and the hip after THA. The native hip was then moved into 5 positions of flexion-extension and abduction-adduction (Table I), for each of which the available range of internal-external rotation was measured. These positions were chosen on the basis of studies of high-dislocation-risk movements^26^ and previous studies of hip instability^14,16,18,22,27,28^. A large slack region was observed after THA, so internal-external rotation torque was applied in 2 steps. First, 1 Nm was applied to rotate the hip through the slack region and tauten ligaments past their toe region. Then, the torque was increased from 1 to 5 Nm over 5 seconds, with the angular measurement made as soon as the machine registered 5 Nm of passive resistance.

**TABLE I:** Summary of the 5 THA-Relevant Hip Positions That Were Tested

Name	Position	Rationale
Ext-abd	Full extension, full abduction	An extreme of hip motion, a position at high risk of posterior impingement, subluxation, and anterior dislocation when the hip was externally rotated
Standing	Neutral flexion-extension, neutral abduction-adduction	A position at risk of anterior dislocation with excessive external rotation; commonly studied in in vitro tests
Heel strike	30° of flexion, neutral abduction-adduction	A control position where instability was not expected
Sitting	90° of flexion, neutral abduction-adduction	A position at risk of posterior dislocation with excessive internal rotation; commonly studied in in vitro tests
Flx-add	Full flexion, full adduction	The other extreme of hip motion, a position at high risk of anterior impingement, subluxation, and posterior dislocation when the hip was internally rotated

THA components were then implanted while keeping the capsule intact (see following section). The range of internal-external rotation in the 5 hip positions was then retested in 9 different configurations: 3 head sizes (28, 32, and 36 mm) with 3 neck lengths (0, +5, and +10 mm, where 0 mm was the restored native, i.e., anatomical, neck length). The testing order for head size was randomized between specimens, whereas the neck was lengthened from 0 mm (first) to +10 mm (last). THA component insertions, component neck lengthening, and head-cup exchanges were performed without trauma to the capsule.

Tests were performed at room temperature, with specimens kept moist with frequent water spray. A nominal compressive force of 10 N angled 20° medially and proximally to the mechanical axis of the femur was applied to the joint to ensure the head-cup contact. This angle replicated the typical loading direction during activities of daily living^29^. Subluxations were free to occur in response to the applied movement and load. After component implantation in THA, for some combinations of head size and neck length, the hip dislocated prior to registering 5 Nm of passive restraint, since the range of rotation greatly exceeded the physiological range of motion. When this happened, the rotation required to generate the 5 Nm of capsular restraint was found by relocating the hip and repeating the test using displacement control (thus preventing superior-inferior femoral head translation), as previously reported^22^. Although this resulted in variation in the applied compressive load, it allowed the hip to be rotated to the 5-Nm end point in these extreme cases, thereby providing a representative measurement for the repeated-measures analyses.

### THA Component Insertion Procedure

**Fig. 3:**
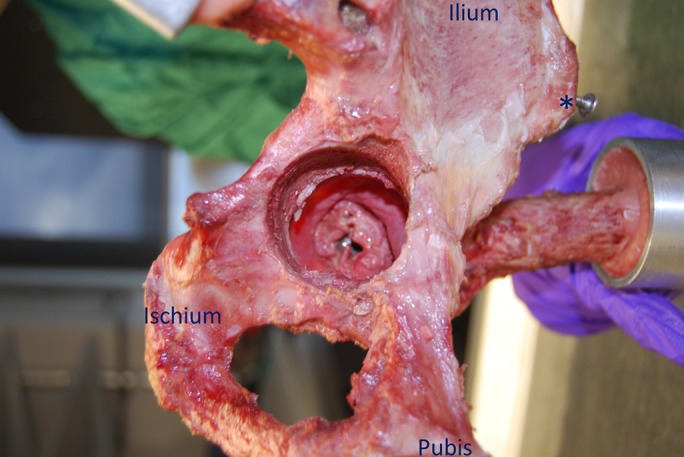
A medial view of a hemipelvis showing an intact hip capsule through the acetabular medial wall with the hip in deep flexion. Regions of the pelvic bone and the anterior superior iliac spine (asterisk) have been highlighted to aid in orientation visualization.

THA component insertion was performed by the senior orthopaedic surgeon (S.K.M.-A.), through an entry portal of the acetabular medial wall, thus protecting and preserving the entire capsule (Fig. 3), similar to the transpelvic technique described by Elkins et al.^15^. This allowed capsular biomechanics after THA to be studied independent of surgical approach.

Femoral component positioning was achieved through a guidewire aligned to the femoral neck (based on the femoral head resurfacing technique). A neck clamp guided the wire from the medial wall, through the mid-fossa, down the neck, and exiting the base of the greater trochanter. The guidewire was enlarged with a cannulated drill, and the central acetabulum was removed with a 40-mm-diameter hole-saw, thus exposing the femoral head. The ligamentum teres was excised and the neck-axis hole enlarged through to the greater trochanter, to accommodate the femoral component while preserving native hip version and varus-valgus alignment. Head-neck resection to the level of the osteotomy in a clinical THA was carried out through the acetabular medial wall opening.

The (specialty) femoral component was based on a thrust plate design (Fig. 2), with a threaded neck-shaft that allowed neck lengthening by screwing from a fixture in the greater trochanter. The head was mounted to the threaded neck with a 12/14-taper connecting component, such that the neck-cup impingement range of motion was identical to that from conventional clinical THA. This thrust plate femoral component was cemented into the greater trochanter. The head was attached to the neck through the medial wall portal.

The acetabular component involved a 28, 32, or 36-mm-diameter bearing with a 170° subtended arc. Given that part of the acetabulum had been removed, the cup was attached directly to the test rig rather than to the pelvis. This rigid attachment held the cup in 20° of anteversion and 45° of inclination (radiographic definition)^30^ relative to the pelvis (Fig. 2). Because the pelvis was in turn rigidly attached to the test rig, this (nonclinical) modality of cup fixation was functionally equivalent to (clinically) rigid fixation of the cup in the pelvis.

Prior to insertion of THA components, 6 small screws were inserted into osseous landmarks to enable 3 repeatable measurements of pelvis-to-femur registration. Those registration measurements were made for 2 hip positions (standing and sitting). Following insertion of the THA components, the native neck length was restored by finding the neck length that minimized the difference between the native and the THA component-implanted condition for these 6 measurements. The remaining error was then used to quantify the accuracy of hip center restoration.

**Fig. 4:**
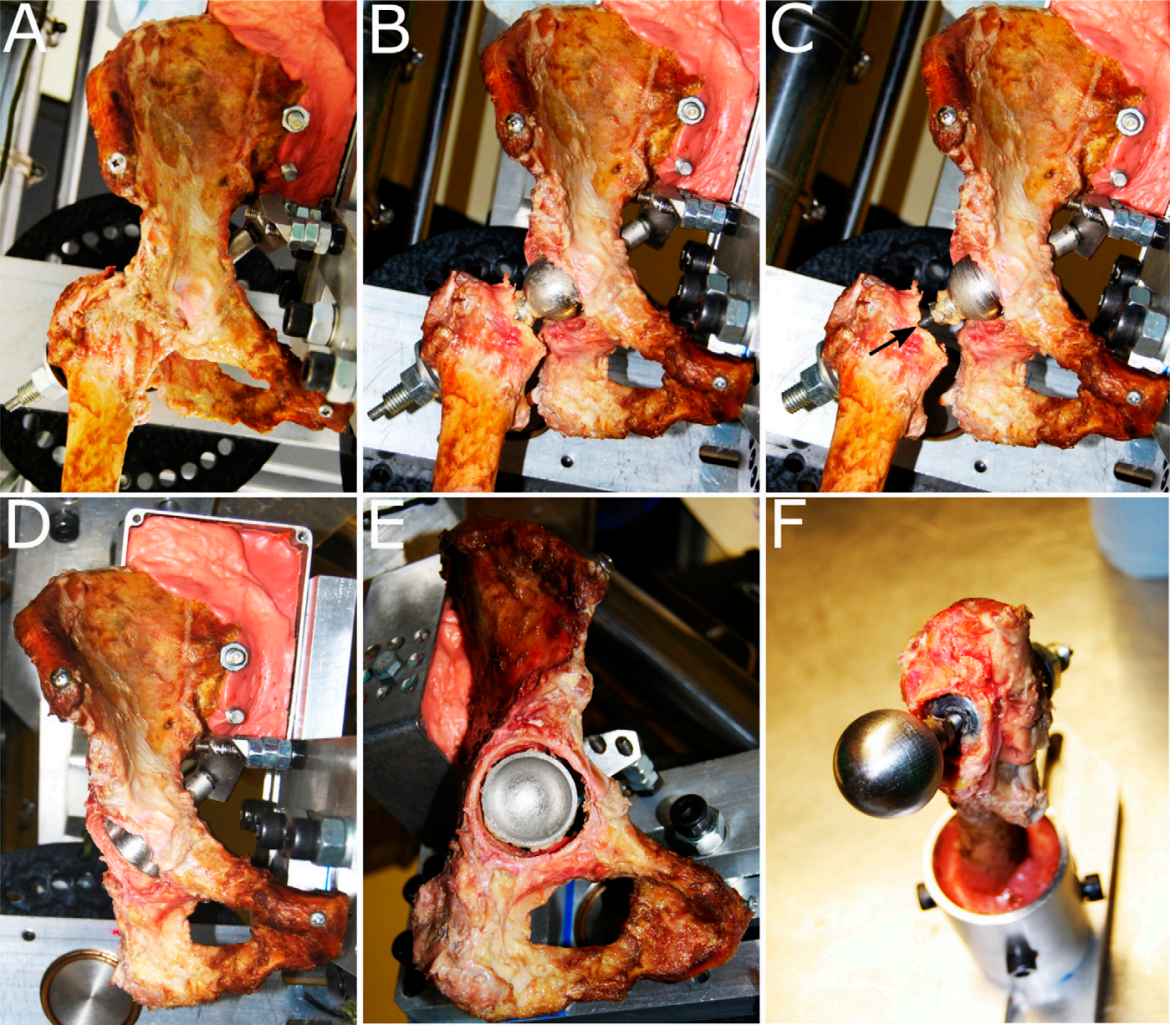
**Figs. 4-A through 4-F** Visual inspection of a hip after THA following testing. **Figs. 4-A, 4-B, and 4-C** An anterior view of the hip in the heel strike position with the capsule intact (**Fig. 4-A**) and resected (**Fig. 4-B**) and with the femoral neck lengthened (black arrow) (**Fig. 4-C**). **Figs. 4-D, 4-E, and 4-F** Inspection at 20° to 45° of cup positioning (**Fig. 4-D**), concentricity of the cup in the acetabulum (**Fig. 4-E**), and the femoral neck osteotomy (**Fig. 4-F**).

The result was experimental THA component implantation with the hip capsule intact and all factors that affected movement axes, range of motion, and implant impingement replicating those for corresponding clinical THA. As a final visual check, following data collection, the capsule was resected, and the reconstruction was inspected to verify that the acetabular component was concentric with the acetabulum, and that the femoral neck had been resected to an appropriate level (Fig. 4).

### Statistical Analysis

Data were analyzed in SPSS (version 22; IBM). Data were tested for normality with a Shapiro-Wilk test. The effects of varying the independent variables (hip position, neck length, and head size) on the dependent variables (range of motion for internal and external rotation) were examined using repeated-measures analysis of variance (RMANOVA). Three analyses were performed:

1. A 3-way RMANOVA using only THA component-implanted data to determine if there was an interaction among head size, neck length, and position.
2. A 2-way RMANOVA examining the effects of hip position and head size with the native hip and 0-mm neck length THA component-implanted data.
3. A 2-way RMANOVA examining the effects of hip position and neck length with native and averaged THA component-implanted head size data.

Post hoc paired t tests with Bonferroni correction were applied when differences across tests (as indicated by the 95% confidence interval) were found. Adjusted p values, multiplied by the appropriate Bonferroni correction factor in SPSS, were reported.

## Results

The functional hip center was restored with an absolute mean error (and standard deviation) of 3 ± 3 mm from native to replaced and 1 ± 1 mm between head exchanges.

**Fig. 5:**
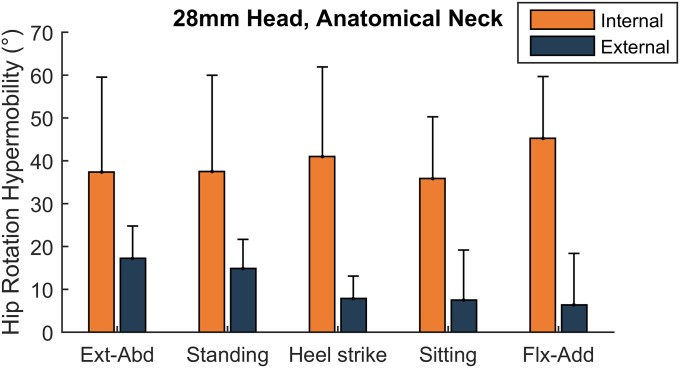
The mean increase in range of rotation compared with the native hip (hypermobility) following THA with 28-mm head size and anatomical neck length. Post-THA, both internal (orange) and external (dark blue) rotation hypermobility was observed, indicating dysfunctional capsular ligaments. Ext-Abd = extension-abduction, and Flx-Add = flexion-adduction. The error bars indicate the standard deviation.

Following THA with an anatomical neck length and 28-mm head size, the hip became hypermobile: the replaced hip rotated beyond the range of the native hip before the capsular ligaments engaged to restrain movement (Fig. 5). Across all positions, the range of internal rotation increased by a mean of 39° (p = 0.003; Table II), which was greater than the 11° increase in the range of external rotation (p = 0.004; Table III).

**TABLE II:** The Mean Change in Internal Rotation with Various Head Sizes*

THA Compared with Intact			THA Compared with THA		
Head Size	Difference† *(deg)*	P Value‡	Comparison	Difference† *(deg)*	P Value‡
28 mm	39 ± 23	0.003§	28 > 32 mm	6 ± 6	0.053
32 mm	33 ± 19	0.002§	28 > 36 mm	8 ± 9	0.074
36 mm	32 ± 16	0.001§	32 > 36 mm	2 ± 7	1.000

* Data are given as the average across all hip positions (main effect). No interaction was detected (p = 0.199); rather, there was a small effect of head size across all hip positions (p < 0.001). For THA compared with intact, positive values indicate the hip was hypermobile after THA. For THA-THA comparisons, the greater-than symbol (>) indicates which head size had the greater range of internal rotation.

† The values are given as the mean and the 95% confidence interval.

‡ T test.

§ The difference was significant (p < 0.05).

**TABLE III:** The Mean Change in External Rotation with Various Head Size*

THA Compared with Intact			THA Compared with THA		
Head Size	Difference† *(deg)*	P Value‡	Comparison	Difference† *(deg)*	P Value‡
28 mm	11 ± 7	0.004§	28 > 32 mm	3 ± 3	0.013§
32 mm	7 ± 6	0.015§	28 > 36 mm	4 ± 5	0.196
36 mm	7 ± 8	0.070	32 = 36 mm	0 ± 4	1.000

* Data are given as the average across all hip positions (main effect). No interaction was detected (p = 0.067); rather, there was a small effect of head size across all hip positions (p < 0.001). For THA compared with intact, positive values indicate the hip was hypermobile after THA. For THA-THA comparisons, the greater-than symbol (>) indicates which head size had the greater range of external rotation.

† The values are given as the mean and the 95% confidence interval.

‡ T test.

§ The difference was significant (p < 0.05).

The effects of head size and neck length on this post-THA hypermobility were examined separately, as there was no 3-way interaction between head size, neck length, and position (p > 0.507), and no 2-way interaction between head size and neck length (p > 0.353).

### Head Size

**Fig. 6:**
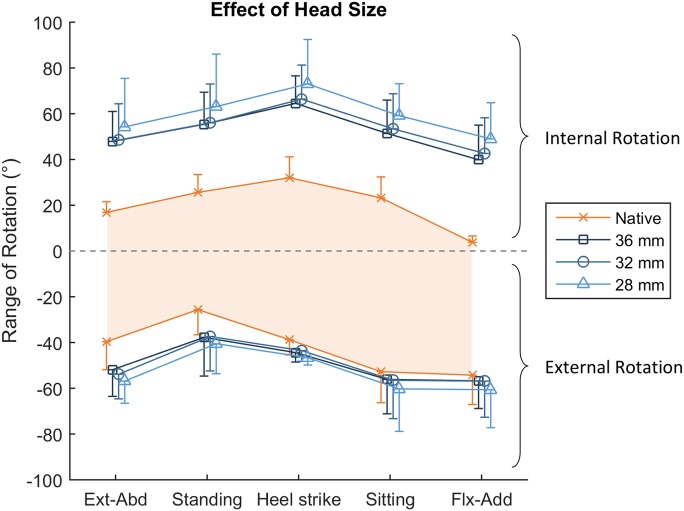
The effect of THA head size on the ability for the capsule to restrain hip rotation with anatomical neck length. Internal rotation is positive, and external rotation is negative. The shaded orange area dictates the measured range of native hip rotation. The graph shows that increasing the head size had a small beneficial effect, resulting in a THA passive restraint envelope that was closer to that of the native hip. Ext-Abd = extension-abduction, and Flx-Add = flexion-adduction. The values are given as the mean, and the error bars indicate the standard deviation.

Larger head sizes did not moderate post-THA hypermobility. Only a small reduction in hypermobility across all hip positions was detected with increasing head size (Fig. 6 and Tables II and III). For internal rotation, all 3 head sizes (with the anatomical neck length) had between 32° and 39° of hypermobility (p ≤ 0.003; Fig. 6 and Table II). For external rotation, the 28 and 32-mm head sizes had 11° and 7° of hypermobility, respectively (p ≤ 0.015; Fig. 6 and Table III), and there was a trend (p = 0.070) for hypermobility with the 36-mm head as well.

### Neck Length

**Fig. 7:**
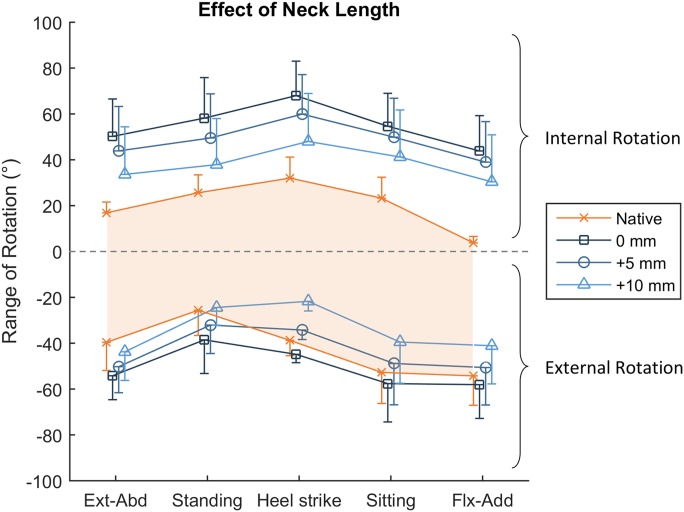
The effect of increasing the neck length on the ability of the capsule to restrain hip rotation. Internal rotation is positive, and external rotation is negative. The shaded orange area dictates the measured range of native hip rotation. Lengthening the neck tautened the hip capsule and reduced the envelope of passive motion, leading to reduced range of external rotation in some hip positions. Ext-Abd = extension-abduction, and Flx-Add = flexion-adduction. The values are given as the mean, and the error bars indicate the standard deviation.

Increasing the THA neck length was able to partially moderate the post-THA hypermobility: for each 5-mm increase in neck length, the passive range-of-motion restraint envelope narrowed by approximately 20°, with the range of both internal and external rotation decreasing by approximately 10° each (p < 0.018; Fig. 7 and Tables IV and V). However, for internal rotation, the anatomical (0-mm) and +5-mm neck lengths continued to have 35° and 28° of hypermobility across all hip positions, respectively (p ≤ 0.013; Fig. 7 and Table IV).

**TABLE IV:** The Mean Change in Internal Rotation with Various Neck Lengths*

THA Compared with Intact†			THA Compared with THA†		
Neck Length	Difference† *(deg)*	P Value‡	Comparison	Difference† *(deg)*	P Value‡
0 mm	35 ± 19	0.002§	0 > +5 mm	7 ± 3	0.001§
+5 mm	28 ± 22	0.013§	0 > +10 mm	17 ± 8	0.001§
+10 mm	18 ± 25	0.207	+5 > +10 mm	10 ± 6	0.002§

* Data are given as the average across all hip positions (main effect). No interaction was detected (p = 0.071); rather, an effect of neck length was detected across all hip positions (p = 0.001). For THA compared with intact, positive values indicate the hip was hypermobile after THA. For THA-THA comparisons, the greater-than symbol (>) indicates which head size had the greater range of internal rotation.

† The values are given as the mean and the 95% confidence interval.

‡ T test.

§ The difference was significant (p < 0.05).

**TABLE V:** The Mean Change in External Rotation with Varying Neck Lengths and According to Hip Position*

THA Compared with Intact			THA Compared with THA		
Neck Length and Hip Position	Difference† *(deg)*	P Value‡	Comparison	Difference† *(deg)*	P Value‡
Extension-abduction					
0 mm	15 ± 10	0.008§	0 > +5 mm	4 ± 6	0.243
+5 mm	11 ± 11	0.063	0 > +10 mm	10 ± 5	0.001§
+10 mm	4 ± 8	0.678	+5 > +10 mm	7 ± 5	0.015§
Standing					
0 mm	13 ± 9	0.007§	0 > +5 mm	6 ± 5	0.014§
+5 mm	6 ± 8	0.124	0 > +10 mm	14 ± 12	0.018§
+10 mm	−1 ± 9	1.000	+5 > +10 mm	8 ± 10	0.131
Heel strike					
0 mm	6 ± 5	0.028§	0 > +5 mm	11 ± 4	<0.001§
+5 mm	−5 ± 5	0.061	0 > +10 mm	23 ± 5	<0.001§
+10 mm	−17 ± 7	<0.001§	+5 > +10 mm	13 ± 6	0.001§
Sitting					
0 mm	5 ± 13	1.000	0 > +5 mm	9 ± 3	<0.001§
+5 mm	−4 ± 15	1.000	0 > +10 mm	18 ± 5	<0.001§
+10 mm	−13 ± 15	0.091	+5 > +10 mm	9 ± 3	<0.001§
Flexion-adduction					
0 mm	4 ± 13	1.000	0 > +5 mm	7 ± 3	<0.001§
+5 mm	−4 ± 16	1.000	0 > +10 mm	17 ± 4	<0.001§
+10 mm	−13 ± 16	0.112	+5 > +10 mm	10 ± 3	<0.001§

* The effects depended on the hip position (p = 0.004): for THA compared with intact, positive values indicate the hip was hypermobile after THA, while negative values imply nonphysiological restriction. For THA-THA comparisons, the greater-than symbol (>) indicates which head size had the greater range of external rotation.

† The values are given as the mean and the 95% confidence interval.

‡ T test.

§ The difference was significant (p < 0.05).

For external rotation, the effect of neck length on the range of rotation varied with hip position (p = 0.004). In all positions of low hip flexion, the anatomical neck length caused hypermobility: by 6° at heel strike, and up to a maximum of 15° in extension-abduction (p ≤ 0.028; Fig. 7 and Table V). Conversely, with the neck lengthened by +10 mm, rotation was nonphysiologically restricted at heel strike by 17° (p < 0.001), and there was a trend for similarly reduced range of external rotation in positions of deep hip flexion (sitting and flexion-adduction, p ≤ 0.12).

## Discussion

This study was a small series using cadavers and, therefore, conclusions should be applied clinically with caution. Even so, our data demonstrated large effects of THA geometry on the function of preserved capsular ligaments in the early postoperative period.

**Fig. 8:**
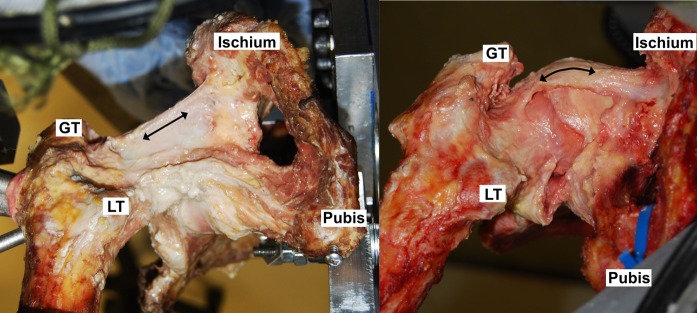
Inferior views of internally rotated hips in a sitting position. Following THA in the present study (left), the ischiofemoral ligament was unable to wrap around the femoral head, indicated by its straight line of action. Increasing the neck length pretensioned the ligament, allowing it to become taut (left), but the passive restraint envelope was not equivalent to the native hip because of the loss of native hip ligament wrapping (right). Black arrows indicate the line of tension in the ligament. GT = greater trochanter, and LT = lesser trochanter. (The image on the right is reproduced, with modification, from: van Arkel RJ, Amis AA, Cobb JP, Jeffers JRT. The capsular ligaments provide more hip rotational restraint than the acetabular labrum and the ligamentum teres: an experimental study. Bone Joint J. 2015 Apr;97-B[4]:484-91. Reproduced with permission of the British Editorial Society of Bone and Joint Surgery through PLSclear.)

The most important finding of this study was that THA inherently reduced the ability of native anatomy capsular ligaments to restrain hip motions. This was because in the native hip, capsular ligaments pull taut by wrapping tightly around the surface of the native head, whereas following THA, the smaller postoperative femoral head does not have this tensioning mechanism. The posterior capsule was most affected after THA, particularly in flexion with internal rotation, because in these positions the ischiofemoral ligament required wrapping to tauten (Fig. 8). The anterior capsule was less affected, particularly in flexion, because it had less of a dependence on wrapping. With its straight line of action, the lateral arm of the iliofemoral ligament, the primary restraint to external rotation^22^, was largely unaffected by the THA procedure to the extent that lengthening the neck too far restricted external rotation. This means that, in the early postoperative period, the native anterior capsule anatomy may remain functional in constraining range of motion, but the native posterior capsule anatomy will not.

Loss in early capsular function after THA could not be moderated through changes in head size, as all common sizes (28 to 36 mm) were considerably smaller than the native femoral head (typically 52 mm in diameter)^31^, but it could be partially moderated through neck length. The implications depend on surgical approach. When THA is performed from the posterior, there is an opportunity to preserve the anterior capsule, which our data showed could remain functional in the early postoperative period. Combined with a typical posterior repair, which removes slack through suturing to holes drilled in the greater trochanter^9,32^ or to the anterior-superior capsule^2^, the capsule could provide short-term benefits. Clinical^2,6,13,17^, in vitro^14,18^, and computational^15^ studies have suggested that this coupling of anterior preservation with posterior repair can improve hip stability and reduce dislocation rates for posterior approaches. When performing THA from the anterior, the posterior capsule can be preserved, which our data suggested would offer little biomechanical benefit without neck lengthening or prior to any longer-term adaptations. Therefore, the early protection against instability associated with the anterior approach (dislocation rate, 0% to 3%)^33-38^ likely comes from other sources, such as dynamic stability from preserved musculature.

The restoration of femoral offset and leg length (affected by neck length) are important THA goals, as they greatly affect the abductor mechanism and gait mechanics^39,40^. We found that capsular biomechanics were also affected by these variables, as varying neck length effectively either pretensioned or slackened the ligaments. Akin to total knee arthroplasty, where soft-tissue techniques for gap balancing find the compromise between range of motion and knee stability^41^, the results of our study suggest that, for the capsular ligaments to have a beneficial role after THA, ligament tensions need to be rebalanced. Capsular function is sensitive to positioning: even a 5-mm change to neck length affected the passive restraint envelope by 20°. This means that after the components have been placed, if neck length was inadvertently shortened, any preserved portions of the hip capsule would be dysfunctional in the early postoperative period. This may help to explain recent clinical findings that a reduction of >5 mm in leg length resulted in an increased dislocation risk^42^. Conversely, if lengthened too far, i.e., ≥10 mm, the anterior capsule may require intraoperative release to relieve nonphysiological restriction of range of motion. An alternative implication of these data is that, when a trial component is placed, a preserved anterior capsule may provide a useful gauge for interpreting whether neck length has been restored, with laxity indicating that the neck may be short, and tightness indicating that the neck is too long.

Testing cadaveric tissue in vitro provided time-zero data that are most applicable in the early postoperative period because of neglect of healing and scar-tissue formation, which could alter the findings of our study. However, the cadaveric model allows experimentation and measurements that may not be ethically possible in live patients, while maintaining realistic anatomical and material properties that may not be realized in other laboratory or computational approaches. Our cadaver data may, therefore, help to interpret clinical findings in the early postoperative period when the risk of hip instability is greatest^35,43-45^ and may help to determine the need for, and value of, lifestyle restrictions and hip precautions, which continue to be keenly debated^34,46-50^. It may also be the case that balancing the capsule appropriately at time zero would initiate appropriate biomechanical conditions for better long-term function, but this would certainly need a clinical project to evaluate. Muscle action and restraint greatly contribute to hip mechanics and stability, but they were not included in this study to isolate the effects of THA on capsular biomechanics. Our medial wall approach further removed bias and confounding factors from these data. It avoided using a specific incision or repair technique, for which there are many in routine clinical use^1-12^, and it allowed for a repeated-measures study design that reduced the effects of interspecimen and procedural variability. The resulting baseline data on the effects of head size and neck length can therefore be interpreted with respect to any surgical approach. The internal rotation results would be most relevant to an anterior or lateral approach that preserves the posterior capsule, while the external rotation results would be more relevant to a posterior approach that preserves the anterior capsule. This study used internal-external rotation to demonstrate the loss in ligament wrapping post-THA; similar trends will apply to flexion, extension, abduction, and adduction movements that are restrained by wrapping in the native hip. Our results may have been influenced by the pseudo-THA components designed for this study, which were necessarily different from commercially available products. However, care was taken to control factors that were previously shown to influence range of motion: acetabular component position and subtended arc, femoral version, neck angle, neck length, and head-neck junction. Our reported errors in restoring the center of rotation were considered acceptable. Femoral version was not formally investigated, but the pilot test for this study (n = 1) found that anteversion pretensioned the anterior capsule, constraining external rotation, and retroversion tightened the posterior capsule, constraining internal rotation. Loading was approximated to an average direction, with a magnitude considerably lower than in vivo (which is often >2 kN)^51^. In vivo loading could serve to change subluxation, by perhaps resisting it, compressing the head more into the joint, or by encouraging it, overcoming capsular resistance and causing dislocation.

Preserving capsular function could offer benefits to patients having THA through preserving the defenses of the native hip against instability. However, this study has demonstrated for the first time, as far as we know, that native anatomy does not allow the posterior capsule to provide a stabilizing function after THA. Increasing neck length to increase tension in the posterior capsule can restore some stabilizing function, but it also affects leg length and the abductor mechanism and risks overtightening the anterior capsule, necessitating capsule release to restore range of motion. A larger head diameter (36 mm) did not alter this finding. The anterior and posterior capsule must, therefore, be treated differently. It is challenging to restore posterior capsular function when the hip is accessed via the anterior capsule, meaning that early postoperative stability must come from elsewhere, such as preserved musculature. However, if the surgical approach is posterior, there is an ideal opportunity to remove slack from the posterior capsule during repair, leaving the anterior capsule intact with an anatomical +5-mm neck length, thus restoring function in the early postoperative period to some extent.
